# Implementation of ‘One Health’ approach in Kerala state, India – A systematic review

**DOI:** 10.1002/vms3.1307

**Published:** 2023-10-17

**Authors:** Mohammed K. Suhail, Dorothy Hannis, Alan Armstrong, Alan Rhodes

**Affiliations:** ^1^ School of Health and Life Sciences Teesside University Middlesbrough UK; ^2^ Moray House University of Edinburgh Edinburgh UK

**Keywords:** emerging zoonotic diseases, One Health, One Health implementation Kerala, systematic review

## Abstract

With humans, animals and the environment being as interconnected as they are, the science describing their interactions needs to cut across disciplinary boundaries. Systems research at the interface between the three goes by several names, such as ‘Eco‐Health’ and ‘Planetary Health’, each with a varied focus, but the concept of ‘One Health’ (OH) has stood out as the most popular one. COVID‐19 has reiterated the importance of OH in response to health challenges. This review aimed to assess the OH approach integration and implementation level in Kerala state, India, in the context of emerging zoonotic diseases. A systematic literature review was conducted by searching for relevant articles with specific keywords across six electronic databases. This involved screening the initial hits for titles and abstracts, then systematic sorting to identify the ones that met the criteria, followed by more thorough scrutiny to finally shortlist the six studies to be included in the review. We found that OH in Kerala has made good progress, as evident from a few recent examples, but has a long way to go with significant challenges. In line with the study's aim, identifying and analysing what is already done, what is missing and what needs to be done can have wider implications for future OH implementation. Relevant threats and opportunities were identified, with lessons for Kerala and India and broader applications.

## Graphical abstract

Identifying and analysing what is already done, what is missing and what needs to be done can have wider implications for future One Health (OH) uptake. This systematic review has identified the relevant threats and opportunities in the implementation of OH in Kerala, with lessons for the state and India and broader applications.

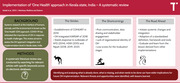

## 1 INTRODUCTION

One Health (OH) is defined as ‘an integrated, unifying approach that aims to sustainably balance and optimise the health of people, animals and ecosystems. It recognises the health of humans, domestic and wild animals, plants and the wider environment (including ecosystems) are closely linked and interdependent’ (Adisasmito et al., 2022) (p. 2). The concept of OH was first envisioned as early as 400 BC in the works of Hippocrates. Greek scholars noted this and adopted the idea of how humans, animals and the environment are connected and need to be in synchrony for overall health, welfare and stability (Roncada et al., 2014).

Population explosion and industrialisation, leading to unchecked deforestation and destruction of natural habitats, have caused the inevitable disturbance in the ecological balance between humans and animals in ways that weren't seen previously. This proliferation, apart from other environmental consequences, has increased the risk of zoonotic diseases around the world (Tazerji et al., 2022). According to the World Health Organization (WHO) (2022a), close to 60% of all emerging infectious diseases (EID) are zoonotic; 75% of over 30 of the new human pathogens detected in the current century are of animal origin (WHO, 2022a). Some well‐known examples are COVID‐19, Middle East respiratory syndrome coronavirus, severe acute respiratory syndrome, influenza, Rift Valley fever, West Nile virus and yellow fever.

The misleading perception that money is being saved by ignoring environmental concerns, animal health, emergency preparedness and health systems strengthening has been paid for at a cost several times over (WHO, 2022b). The COVID‐19 pandemic has undeniably illustrated how closely people, animals and the environment are interconnected. Although the mechanism that caused the spill‐over of the virus from its animal reservoir is still not entirely unravelled, it has again underlined the OH strategy's importance. Thus, there is currently more interest in applying and putting this strategy into practice by public health professionals, even though the OH concept is not new and has long been at the heart of multi‐sectorial and multidisciplinary conversations globally.

In the modern era, American veterinary epidemiologist Dr. Calvin Schwabe first highlighted in 1964 the need for human and animal health professions to come together to tackle zoonotic diseases (Atlas, 2012). The present worldwide OH movement was sparked by the 2004 Manhattan Principles, which are a set of recommendations for building a comprehensive approach to preventing epizootic and epidemic zoonotic diseases and preserving ecological balance (OneWorldOneHealth.org, 2004). A formal ‘One Health Initiative Task Force’ was then set up in 2008, jointly by the American Medical Association, the American Public Health Association and the American Veterinary Medical Association. The task force aimed to address issues such as emerging zoonotic diseases and antimicrobial resistance through the OH concept and broader objectives such as disaster preparedness and response (Nolen, 2007). In May 2021, the interdisciplinary One Health High‐Level Expert Panel (OHHLEP) was established with the cooperation of the WHO, the World Organization for Animal Health, the Food and Agriculture Organization (FAO) and the United Nations Environment Programme (UNEP). The intention to advance the idea of OH with policies and appropriate actions was evident in the founding of OHHLEP, which also shows that the urgency and complexity were acknowledged at the highest level.

India was initially not far behind in adopting OH. A long time before the first H5N1 case hit the shores of India in early 2006, the action plan for its containment by the Department of Animal Husbandry, Dairy and Fisheries and the Directorate General of Health Services contingency plan for the management of human cases were drawn up in 2005 (Nagarajan et al., 2012). After a long relative hiatus, with much deliberation, the government of India held a consequential dialogue in 2019 with the relevant ministries to draft a national OH policy. However, there has again been only incremental progress since. On the regional stage, the ‘Delhi Declaration’ was signed by the member states of WHO Southeast Asia in the same year, agreeing to implement inter‐sectorial collaboration following the OH approach for emergency preparedness and response (WHO, 2019).

Kerala state, with a population of 33.4 million (Census India, 2011), boasts high literacy rates, relatively low birth and death rates and maternal and infant mortality rates similar to high‐income countries. It also has a nearly 100% infant immunisation rate, decreasing infant mortality and vaccine‐preventable diseases (Journals of India, 2021). These comparatively higher indices were attained despite the restraints of low–middle‐income countries and low per capita income. The state's development model is frequently cited and studied in research and academia, commonly referred to as the ‘Kerala Model’ (Veron, 2001).

This model health care system has faced new challenges in recent years, arising from various factors. A resurgence of numerous communicable diseases, such as falciparum malaria and dengue, has been noticed by the state's IDSP (Integrated Disease Surveillance Programme), which are quite often underreported. Newer zoonotic diseases like Kyasanur Forest Disease (KFD), Nipah, Lyme disease and scrub typhus are emerging. Studies surmise that the rapid influx of migrant workforce from other states of India is causing the seeding of endemic diseases from those areas, such as malaria and cholera in Kerala (Sukumaran & Pradeepkumar, 2015). Unplanned and unchecked urbanisation is rampant, leading to an extra load on the urban health care system and the subsequent menace of urban waste and sewage. This pollutes the water bodies and the surrounding areas of the landfills, disturbing the ecosystems (Praveen Lal & Nair, 2017). Traditional zoonotic diseases like rabies, brucellosis and anthrax continue to pose a significant risk in Kerala. Newer zoonotic diseases like leptospirosis have become endemic in many districts, whereas others have shown early warning signs of emergence (Sukumaran & Pradeepkumar, 2015). There are similar concerns in mammal and bird health, with diseases and outbreaks being reported regularly.

The state is now experiencing an epidemiological shift; vaccine‐preventable diseases are in check, but other outbreak/epidemic‐prone communicable and non‐communicable diseases have emerged as significant public health challenges. The above‐discussed issues and their underlying causes are entrenched with each other in a complicated way, along with the other determinants of health. This complexity advocates that new‐age challenges require transdisciplinary approaches, which bring together a wide array of current disciplines and technologies. The most optimal solution is strategies for prevention and control, surveillance of both animal and human reservoirs and sharing infrastructural facilities like laboratories through transdisciplinary coordination of OH. Reviewing and summarising the existing literature on the level of implementation of the OH approach in Kerala state will help in drawing lessons on the importance of OH and strategies for effective implementation for other states and countries and aid Kerala in improving the integration of OH by learning from the current strengths and weaknesses.

## 2 MATERIALS AND METHODS

**FIGURE 1:**
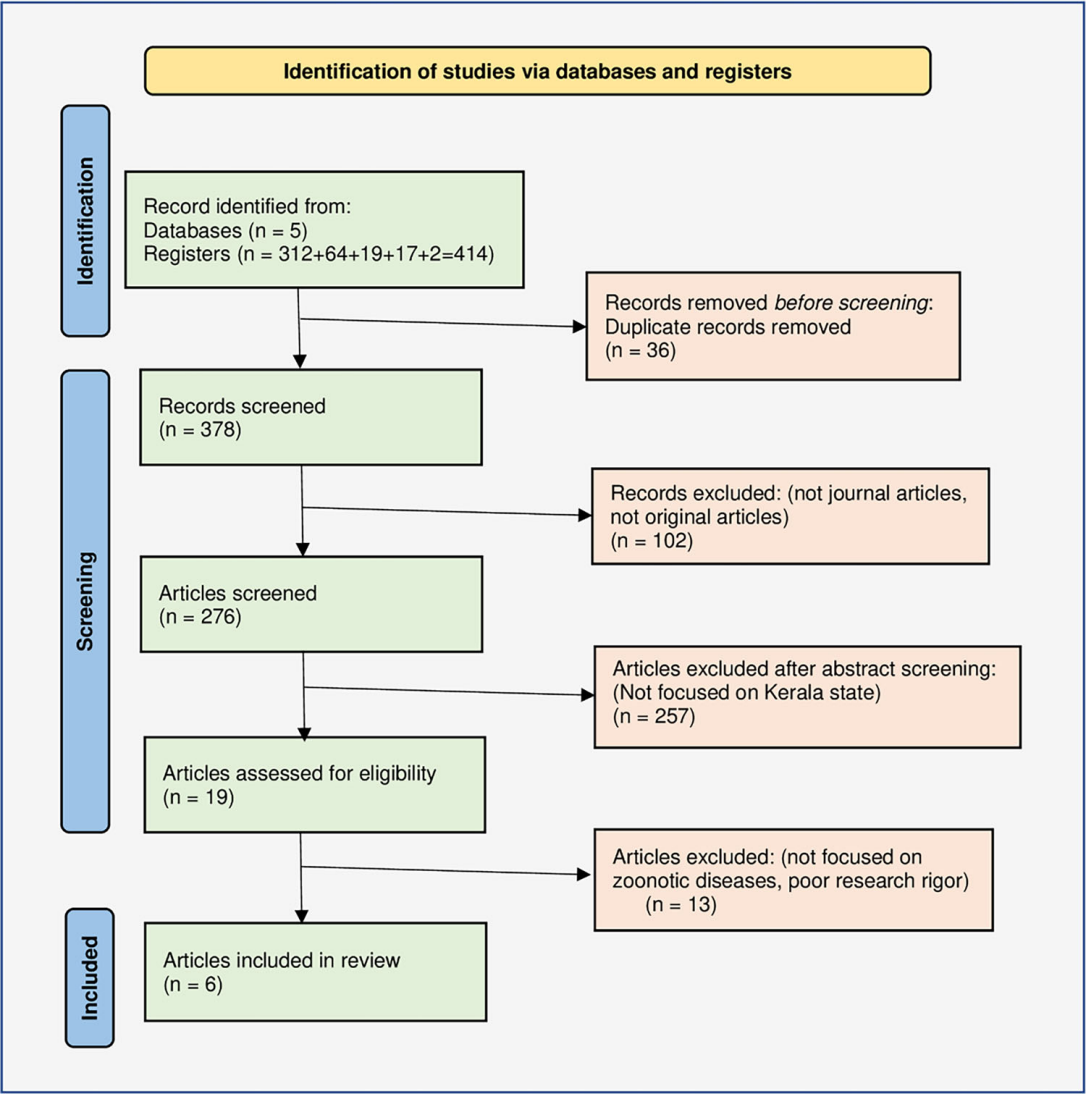
Prisma flowchart of the literature search (Page et al., 2021).

A systematic search was conducted across the five most relevant public health databases: Scopus, EMBASE, Web of Science, PubMed and CINAHL, and the results are reported according to the PRISMA format for systematic reviews (Page et al., 2021). The main search keywords deployed were ‘Kerala’ and ‘One Health’, and an advanced search was conducted using the combination of these keywords, Boolean Operators and search filters. Due to a low number of initial hits, the search was not limited by the publication year, research design or any other criteria except the English language. Initial results were first de‐duplicated and then filtered for non‐peer‐reviewed journal articles to be removed. This was followed by screening abstracts for relevance and assessment for eligibility and then reviewing the full text, leading to six studies being chosen for the final review (Figure 1).

**FIGURE 2:**
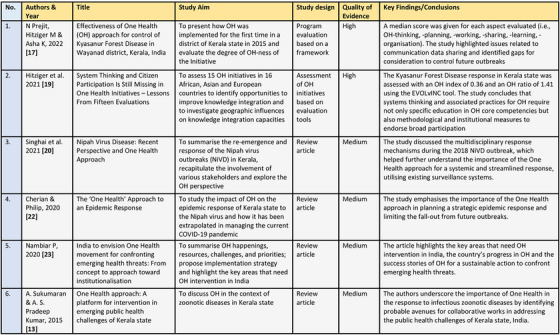
Summary of the included studies.

The predetermined inclusion–exclusion criteria were applied, the purpose of which was to pick the most relevant articles and eliminate extraneous ones specifically. Studies on OH in the context of Kerala state with full‐text available in English were considered for inclusion. Articles not directly discussing aspects of OH within a zoonotic context in Kerala and ones with poor research rigour were excluded. Grey literature was also searched for relevant publications, but nothing reportable was found. The framework Caldwell et al. (2011) proposed for critiquing health research was used to assess the quality of the included papers. Data and information were extracted from the included studies and reviewed for recurrent themes and relevant findings (Figure 2) in the context of this review. The extracted results were analysed and led to three broad themes, discussed in the next section.

## 3 RESULTS

The systematic review reveals that overall, there is a paucity of high‐quality research studies around OH integration and implementation in Kerala state, especially in the context of emerging and re‐emerging zoonotic diseases. This is partly because OH is a relatively new concept and has only gained momentum in India and Kerala in recent years. In line with the aims of this review, the findings from the included studies can be discussed under three broad themes: The strides – what has already been achieved; the shortcomings – what is missing and the challenges; and the road ahead – what needs to be done. Each of the themes and other general findings are presented here in detail.

### 3.1 The strides

This theme highlights the different areas where there has been progress on the OH front in the Kerala state, based on the findings of the review. The Centre for One Health Education, Advocacy, Research and Training (COHEART) was established in 2014 in the Wayanad district of the state. The centre's vision is to facilitate the understanding and application of the OH concept by generating and sharing scientific knowledge. COHEART conducts various activities involving government and private organisations from different disciplines to promote inter‐sectorial coordination for OH (Prejit et al., 2022). Kerala was the pioneer in developing an action plan for AMR at the state level in India. An initiative based on the OH principles, Kerala's Antimicrobial Resistance Strategic Action Plan of 2018 was a noteworthy step in setting up a model for OH uptake (Ranjalkar & Chandy, 2019). Other examples of effective OH integration in the management of zoonotic diseases in Kerala include KFD, influenza A (H5N1) and Nipah (NiV).

Using standardised aspect‐specific assessment tools, Prejit et al. (2022) evaluated six OH facets of the KFD response programme in the state: thinking, planning, working, sharing, learning and systemic organisation. These were scored based on an extensive survey questionnaire and other formative methods, such as interviews, field visits, workshops and discussions. The OH‐ness was scored low in planning, sharing and learning, medium in thinking and high in working and organisation. Overall, the response to KFD in Kerala had a robust and institutional approach grounded in key OH applications and sustainability. The implementation involved multi‐sectorial collaboration and seamless coordination of numerous governmental departments. In the other study by Hitziger et al. (2021: 11), the KFD response in Kerala state was scored with an OH index of 0.36 and OH ratio of 1.41 based on the ‘evaluating knowledge integration capacity in multi‐stakeholder governance’ (EVOLvINC) tool. It was observed that the OH knowledge integration capacity was the highest during the project implementation stage and lowest during the evaluation stage (Hitziger et al., 2021).

In the 2018 Nipah outbreak in Kerala, the importance of the OH approach was highlighted due to the multidisciplinary collaboration and utilisation of existing surveillance systems in delivering a streamlined response to the outbreak. The lessons learned from 2018 were evident, as no secondary cases were reported after the detection of the index cases in the subsequent outbreaks of 2019 and 2021 (Singhai et al., 2021). The veterinarians made significant contributions to the response efforts by tracking the source and spread of the virus. The Animal Husbandry Department supported the response by providing valuable inputs about the local ecosystems and habitats of bats and by spreading general awareness in the affected areas. For example, due to the perceived risk of spread, the local people attempted to clean and clear out their water wells – the common roosting spots for bats (Kumar & Kumar, 2018). Forcing them out was bound to worsen an already high‐risk situation, and timely and targeted educational interventions from the government departments minimised this activity. The central government formed a multi‐disciplinary team chaired by the Union health minister, led by the National Centre for Disease Control, with other government departments and experts as members. They assisted the state and local teams with technical inputs, training, logistical support and other activities, such as contact tracing, case detection and ecological surveys (Cherian & Philip, 2020).

Nambiar (2020) noted that good multi‐sectorial collaboration was also demonstrated in the 2015 H5N1 outbreak in the state. To deal with the bird hosts, including chickens, ducks and other birds within a 1 km radius of the affected area, the Animal Health Department coordinated with other pertinent departments and planned and carried out their culling. They set up a 24‐h control room, provided personal protective equipment to the general public and held awareness campaigns. Furthermore, surveillance in a 10 km radius, tracking of dead birds in the area and expanded monitoring in the surrounding districts for migratory birds were also implemented. Similarly, the health department carried out disease awareness campaigns, frequent health examinations of poultry workers, biosecurity measures and provision of personal protective equipment. The local media provided updates on the situation and awareness about the risks and preventive measures, and the food safety department issued guidelines for the safe use of poultry products. The Forest Department assisted in the timely reporting of migratory birds’ deaths and carrying out their autopsies (Nambiar, 2020). These findings and accounts underscore the initiatives and success stories of OH in Kerala state. Although not at an ideal level, there is good progress in the right direction.

### 3.2 The shortcomings

The previous theme discusses what has been achieved; this one puts forward what is missing and the challenges. Contextual considerations, organisational flexibility at the institutional level, a solid methodological foundation and proficient practitioners at the project level are the prerequisites for starting any initiative, including OH. Having these in place is essential for its success; however, it has been frequently reported that initiatives are implemented even when these conditions are poorly met (Regeer & Bunders, 2009). Prejit et al. (2022) underscored similar crucial shortcomings in the context of KFD and OH, such as gaps in communication, data sharing and learning and lack of involvement of broader stakeholders. Low scores for planning, sharing and learning and a medium score for thinking were given to the OH‐ness by their study. Hitziger et al. (2021) noted that the evaluation stage was when the OH knowledge assimilation capacity was at its lowest. They further allude to stakeholders and users were not well acquainted with the tools and evaluation methods using the systems approach. However, the stakeholders acknowledged the utility of the new approach and its long‐term benefits.

India is deemed as one of the significant global hubs for diseases by experts (Kessler & Peterson, 2017). Although individual disease surveillance programmes at the human and animal levels, such as the IDSP and the National Animal Disease Reporting System, have been successful to a reasonable extent in recent years, they falter in regard to tracing the chain of transmission at the zoonotic interface (Nambiar, 2020). Another glaring gap is the lack of institutionalisation and missing organisational identity of OH. Overall, inter‐sectorial communication and coordination, not just concerning OH, has also hitherto been a significant area of concern in India, especially with the governmental departments (Nambiar, 2020). Evaluation of contextual risks, identification of driving factors of disease emergence and actionable knowledge derived from multisource complex data are vital to shielding communities from potential threats. As reported above, these factors were missing or noticeably weak in some of the studies.

The broader literature posits that multi‐sectorial partnerships such as OH solicit the involvement of policymakers, who are bound by the framework of their purview and scope of work, and this can be challenging, especially in low and middle‐income countries such as India. Greater adoption and use of collaborative concepts are impeded by their unwillingness to widen their perspective. Some studies contend that this fundamentally conservative mindset affects how mainstream OH initiatives fare since experts are often limited by the pursuit of self‐centred and fixed objectives, which results in reductionism and fragmentation (Lee & Brumme, 2013). Such seclusion can be detrimental in the face of challenges, especially for emergency preparedness and response. In summary, there are many deficiencies in the integration and implementation of OH in Kerala, and acknowledging and addressing them is critical for improvement.

### 3.3 The road ahead

This theme presents the way forward for the OH approach in Kerala, exploring what can be and needs to be done to improve OH integration and implementation. An extensive systematic review by dos Ribeiro et al. (2019) has noted that the development of OH programmes continues to face several difficulties. Despite the fact that various OH solutions have been put forth, most have only been implemented in local contexts and at more minor levels. The OH approach's effectiveness also depends on changes in systemic, institutional, cultural and social behaviours, and not just local initiatives (dos Ribeiro et al., 2019). Merely assembling a team of diverse specialists is insufficient to induce a paradigm change for resolving global health issues. Stakeholder and community engagement by combining their ‘real world’ experience with knowledge co‐creation is also needed.

The OHHLEP's operational definition of OH seeks to be comprehensive, to foster better understanding across disciplines and sectors and to aid the concerned countries and organisations in formulating their programmes, strategies and implementation plans. The beneficiary of this includes the recently released Joint Plan of Action for One Health (2022–2026) – a crucial strategic framework that will direct the cross‐sectorial cooperation efforts of the WHO, OIE, FAO and UNEP (Adisasmito et al., 2022). A wide range of other organisations and projects, such as national/regional secretariats and local initiatives, could also greatly benefit from it. OHHLEP's definition should be viewed as a broad collection of guiding concepts that may further be customised for various contexts and stakeholders.

Hitziger et al. (2021) concluded that systems thinking and associated practices for OH require not only specific education in OH core competencies but also methodological and institutional measures to endorse broad participation. The key areas they identified for improvement in OH implementation are considering systemic characteristics, engaging external stakeholders, knowledge sharing and collective learning. Singhai et al. (2021) underscored the need to integrate and institutionalise the OH approach in responding to zoonotic diseases and public health emergencies in general. The assimilation of the OH concept and multi‐sectorial coordination displayed during the NiV outbreaks in Kerala needs to be expanded and implemented at all levels: national, state and district. As discussed earlier, gaps in communication and data sharing and learning, and lack of involvement of broader stakeholders were identified as significant barriers to this (Prejit et al., 2022).

**FIGURE 3:**
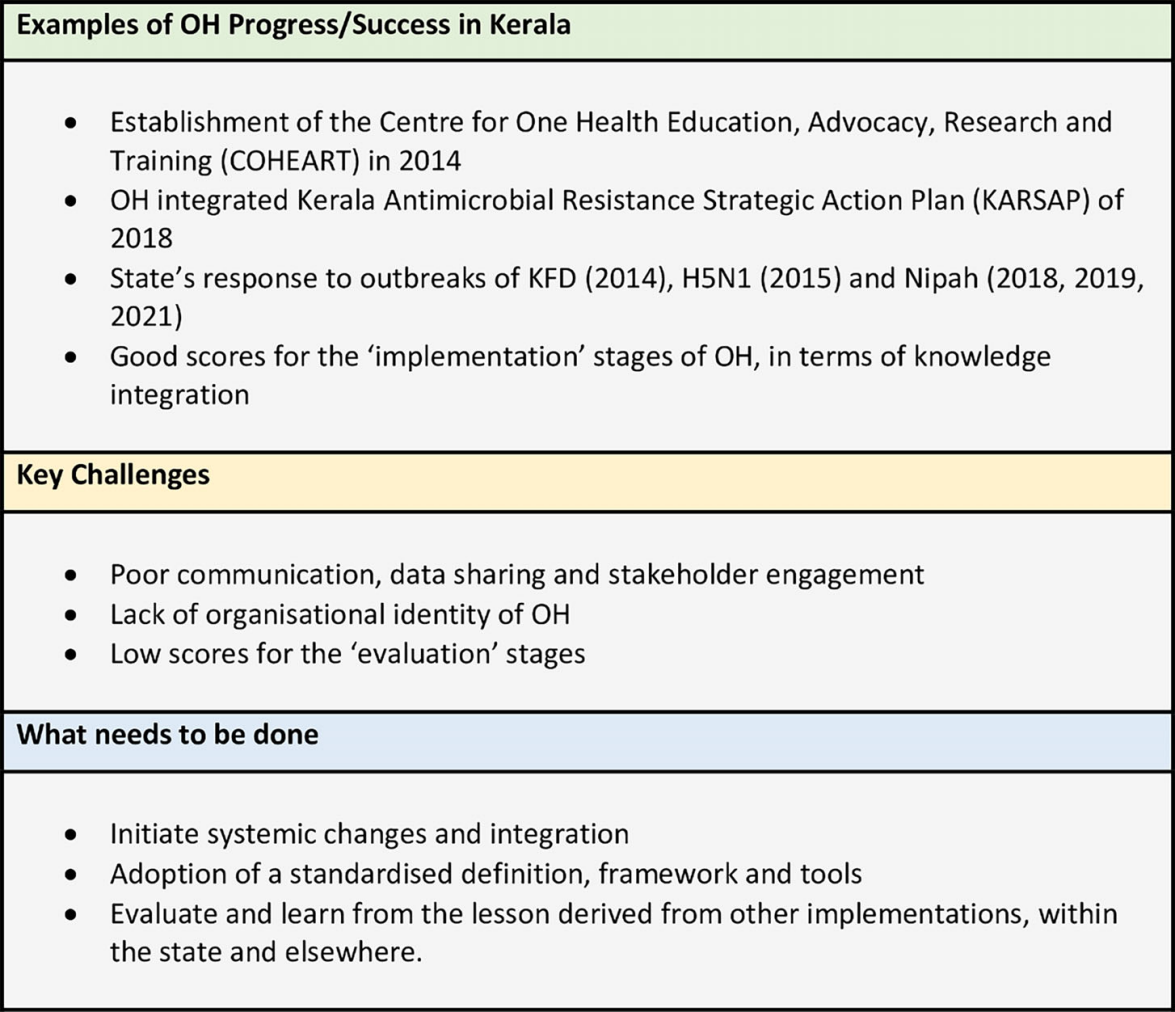
Key points from the findings.

In addition to the above, critical suggestions put forth by Prejit et al. (2022) to improve OH outcomes in Kerala include enhancing surveillance systems and incorporating essential tools (such as NEOH) from the outset. All these must be incorporated into the strategic framework for effective implementation of OH (Bhatia, 2019). Some of the areas where OH can intervene, apart from zoonotic diseases, are, for example the prevention of food safety hazards. This can be achieved through the surveillance and investigation of foodborne illness outbreaks, laboratory networks for pathogen identification, collaborative research, improving animal health to help reduce food contamination by infectious agents and training and awareness programmes on food safety measures (Sukumaran & Pradeepkumar, 2015). Figure 3 presents a snapshot of the overall findings of the systematic review.

## 4 DISCUSSION

To fathom the intricacies of transdisciplinary work, the pyramid description of the level of coordination of the disciplines proposed by Max‐Neef (2005) is very handy. At the base of the pyramid is the ‘what exists’ in many specialised fields. The next level is ‘what are we capable of doing’, where the knowledge from the base level is used by specialist practitioners. This is followed by the ‘what is it that we want to do’ level, informed by the previous two levels and guides the inquiry and research process. ‘How should we do what we want to do’ sits at the top of the pyramid and represents the coordination between the stakeholders at all levels of the pyramid (Max‐Neef, 2005). This concept can be applied to the question, ‘How can the OH strategy minimise the risk of new zoonotic diseases in the future’? A transdisciplinary team effort with scientists from medicine, veterinary sciences, ecology, economics and policymakers would be the best bet to address the challenges at all pyramid levels and find OH‐integrated sustainable solutions.

Cherian and Philip (2020) presented the following general recommendations for improved implementation of OH: (1) broaden the representation of OH in India and involve more stakeholders; (2) consider the local context and develop region‐specific but evidence‐based guidelines for tackling zoonotic disease outbreaks; (3) streamline information sharing, media communications and public engagement by setting up a ‘Communications Committee’; (4) explore the development of mathematical models for common zoonotic diseases; (5) identify and monitor large high‐risk settings for human and animal interactions, such as regional and seasonal livestock markets; (6) promote and fund more research and training in the OH domain; (7) reiterate the need to focus on and achieve the broader global aims of reducing carbon emissions and shifting to green resources to minimise the effects of global warming.

At the country level, India has a lot of ground to cover regarding OH; neighbouring nations like Bangladesh have dedicated ‘OH Secretariat’, whereas Bhutan and Nepal are on track to set up one. Its response to EIDs has been far from ideal in lacking a strong policy context. Along with developing a framework for monitoring and evaluating the OH programme, increased budgetary spending for the human, animal and environmental health sectors is needed (Chatterjee et al., 2016). Institutionalising OH governance should be the next rational step for OH integration in India. For this, Nambiar (2020) suggested the following steps: establish an inter‐ministerial advisory body, create an OH Steering Committee, draft and endorse a national policy, charter the terms of reference and set up an OH secretariat. India's future policy aims to build an institutional OH platform at the national level and strengthen public health systems, including surveillance and laboratories (Nambiar, 2020).

This review found that collaborated efforts from the concerned sectors have notably helped in the early detection of KFD circulation, good response to Nipah virus and management of other outbreaks in Kerala (Prejit et al., 2022). Although the KFD response highlights the potential of implementing OH in all other PH emergencies, the realisation of this vision is faced with significant challenges, including fragmented strategies across sectors and gaps in communication, among others (Nambiar, 2020). However, the findings also show that governmental and non‐governmental bodies are now increasingly focusing on transdisciplinary cooperation for policy development and decision‐making in response to zoonotic diseases. Coordination among the human, animal and environmental health domains is crucial, and significant changes in the approach are needed in research, policies, governance and systems. It is imperative for effective trans‐disciplinary and multi‐sectorial interaction, collaboration and coordination for adopting and integrating the OH approach (Dente et al., 2022).

The basics of OH have been well recognised and appreciated, but the actual implementation is fundamental to the concept of OH. Its integration was primarily constrained to a few applications for a long time (Vandersmissen & Welburn, 2014). Now, around the world, the OH strategy is successfully being incorporated by several international networks. The recently established South Asia One Health Disease Surveillance Network (SAOH‐Net) comprises representatives from governmental and non‐governmental entities from the fields of food safety, wildlife protection and human and animal health from eight South Asian nations (Yasobant et al., 2019). Technical collaboration and knowledge sharing of the planned OH Secretariat in India with SAOH‐Net will be invaluable. Inspiration can also be drawn from the ‘Family Health Support Centres’ (NASF) under the Unified Health System of Brazil, which have diverse multidisciplinary teams of medical specialists, allied health specialists, alternative medicine practitioners and veterinarians. The NASF was created to enhance the efficiency and case‐resolution capacity of primary health care (da Silva et al., 2012).

Another essential aspect to ensure OH uptake and sustainability is community engagement. Community representatives must be onboarded in OH initiatives, who can contribute with local experience and expertise and assist in sentinel surveillance (Henley et al., 2021). Furthermore, a cost‐effectiveness evaluation framework should be designed in collaboration with nations with OH policies already in place, with customisable components to adapt to regional contexts. Targeted operational research could also aid in the evaluation of the sustainability and effectiveness of OH programmes in prevention and preparedness (Fung & Fu‐Chun, 2021). There is an urgent need to persuade governments to allocate sufficient finances for capacity building on prevention and preparedness, using transdisciplinary and multi‐sectorial strategies to guide appropriate actions. The concepts of ‘interoperable databases’ and ‘data democratisation’ have been proposed as crucial components of the suggested prevention‐preparedness collaboration system since they can promote information sharing (Dente et al., 2022).

Limitations of the study include the lack of homogeneity of the studies due to the limited number that met the criteria and the omission of literature other than in English.

## 5 CONCLUSION

In conclusion, the OH approach has a much broader utility than just zoonotic disease and AMR, from health promotion, improvement and prevention to the identification, readiness, response and recovery from health emergencies. It can be integrated at all levels, including local, state, national, regional and international. Effective implementation of an integrated OH approach depends on political leadership and commitment, along with setting priorities and equitable resource allocation. Further ownership of implementation is also needed at the sectorial, organisational and individual levels. This is hindered by pronounced political, legal, ethical, economic and social factors. Human, animal and environmental health and the resolution of other issues like climate change, biodiversity loss, air pollution and food and water security are at stake. The OH strategy offers a definitive framework for integrating social and environmental protection and supporting resilient and sustainable economic growth (Adisasmito et al., 2022).

A coordinated ground‐level approach to studying and implementing the OH approach must be fostered through engagement with communities led by health experts, veterinarians, environmentalists and anthropologists. Community involvement is essential in determining what is likely to be effective in their environment, which, down the line, impacts surveillance, reporting methods and policies (Durrance‐Bagale et al., 2021). The COVID‐19 pandemic has brought to the fore the importance of preparedness and collaboration against zoonotic diseases, and there can be no better time to bolster the need for uptake and integration of the OH approach, as governments, policymakers and people are willing to listen (Suhail & Moinuddin, 2023). The formulation of policies and enactment into law will require significant political will and effort, together with the onboarding of all stakeholders.

## AUTHOR CONTRIBUTIONS

**Mohammed K. Suhail**: Conceptualization; formal analysis; methodology; resources; validation; visualization; writing – original draft; writing – review and editing. **Dorothy Hannis**: Conceptualization; methodology; supervision; writing – review and editing. **Alan Armstrong**: Supervision; writing – review and editing. **Alan Rhodes**: Supervision; writing – review and editing.

## CONFLICT OF INTEREST STATEMENT

The authors declare no conflicts of interest.

### PEER REVIEW

The peer review history for this article is available at https://www.webofscience.com/api/gateway/wos/peer‐review/10.1002/vms3.1307.

## Data Availability

None
